# MicroRNA Dysregulation in Parkinson’s Disease: A Narrative Review

**DOI:** 10.3389/fnins.2021.660379

**Published:** 2021-04-30

**Authors:** Yong Hui Nies, Nor Haliza Mohamad Najib, Wei Ling Lim, Mohd Amir Kamaruzzaman, Mohamad Fairuz Yahaya, Seong Lin Teoh

**Affiliations:** ^1^Department of Anatomy, Faculty of Medicine, Universiti Kebangsaan Malaysia Medical Centre, Kuala Lumpur, Malaysia; ^2^Department of Biological Sciences, School of Medical and Life Sciences, Sunway University, Selangor, Malaysia

**Keywords:** microRNA, non-coding RNA, Parkinson’s disease, neurodegenerative disease, biomarker

## Abstract

Parkinson’s disease (PD) is a severely debilitating neurodegenerative disease, affecting the motor system, leading to resting tremor, cogwheel rigidity, bradykinesia, walking and gait difficulties, and postural instability. The severe loss of dopaminergic neurons in the substantia nigra pars compacta causes striatal dopamine deficiency and the presence of Lewy bodies indicates a pathological hallmark of PD. Although the current treatment of PD aims to preserve dopaminergic neurons or to replace dopamine depletion in the brain, it is notable that complete recovery from the disease is yet to be achieved. Given the complexity and multisystem effects of PD, the underlying mechanisms of PD pathogenesis are yet to be elucidated. The advancement of medical technologies has given some insights in understanding the mechanism and potential treatment of PD with a special interest in the role of microRNAs (miRNAs) to unravel the pathophysiology of PD. In PD patients, it was found that striatal brain tissue and dopaminergic neurons from the substantia nigra demonstrated dysregulated miRNAs expression profiles. Hence, dysregulation of miRNAs may contribute to the pathogenesis of PD through modulation of PD-associated gene and protein expression. This review will discuss recent findings on PD-associated miRNAs dysregulation, from the regulation of PD-associated genes, dopaminergic neuron survival, α-synuclein-induced inflammation and circulating miRNAs. The next section of this review also provides an update on the potential uses of miRNAs as diagnostic biomarkers and therapeutic tools for PD.

## Parkinson’s Disease

Parkinson’s disease (PD) is the second most common neurodegenerative disorder after Alzheimer’s disease. The clinical signs of PD involved dopamine (DA)-related motor symptoms such as bradykinesia, postural instability, resting tremor, walking and gait difficulties (Jellinger, 2015). Compelling evidences relating to the multisystem effects of PD reported that the primary feature defects in movement control are caused by the death of DA neurons within the substantia nigra pars compacta (SNpc) (Chen et al., 2019). In addition to the motor symptoms, PD patients also presented with other non-motor symptoms which may precede the development of motor symptoms such as olfactory dysfunction, autonomic dysfunction (respiratory, cardia, gastrointestinal, urogenital), sensory symptoms, sleep disorder, neuropsychiatric symptoms (cognitive, mood, dementia, hallucinations) and others (Azmin et al., 2014; Jellinger, 2015). Most PD patients exhibit impaired cognitive function, particularly the ability to concentrate, executive functioning, and experiencing varying degrees of dementia (Goedert et al., 2013; Irwin et al., 2013).

The incidence of PD is between 10 and 50/100,000 person/year, and the prevalence in the population is between 100 and 300/100,000 (Pringsheim et al., 2014). The prevalence and incidence rates of PD among the Eastern population are slightly lower compared to the Western population with a 51.3 – 176.9/100,000 prevalence rate. Genetic and environmental factors could be the reasons to explain a lower PD prevalence rate observed in Asia countries (Abbas et al., 2018). The number of individuals with PD continues to grow from 4.6 million in the year 2005 to an estimate of 9.3 million in 2030 in Western Europe (Dorsey et al., 2007). From the 6.1 million individuals worldwide diagnosed with PD in 2016, 2.9 million (47.5%) were women and 3.2 million (52.5%) were men with a ratio of 1: 1.5 (GBD 2016 Parkinson’s Disease Collaborators, 2018). The differences could be due to the neuroprotective role of estrogens in women, frequent occupational exposures in men and X-linked genetics factors. Aging is one of the known risk factors of PD. This is supported by a study in Olmsted County, Minnesota whereby an increased incidence rate of parkinsonism and PD was reported from 1976 to 2005, particularly in men of age 70 and above (Savica et al., 2016).

### Pathogenesis of Parkinson’s Disease

**FIGURE 1:**
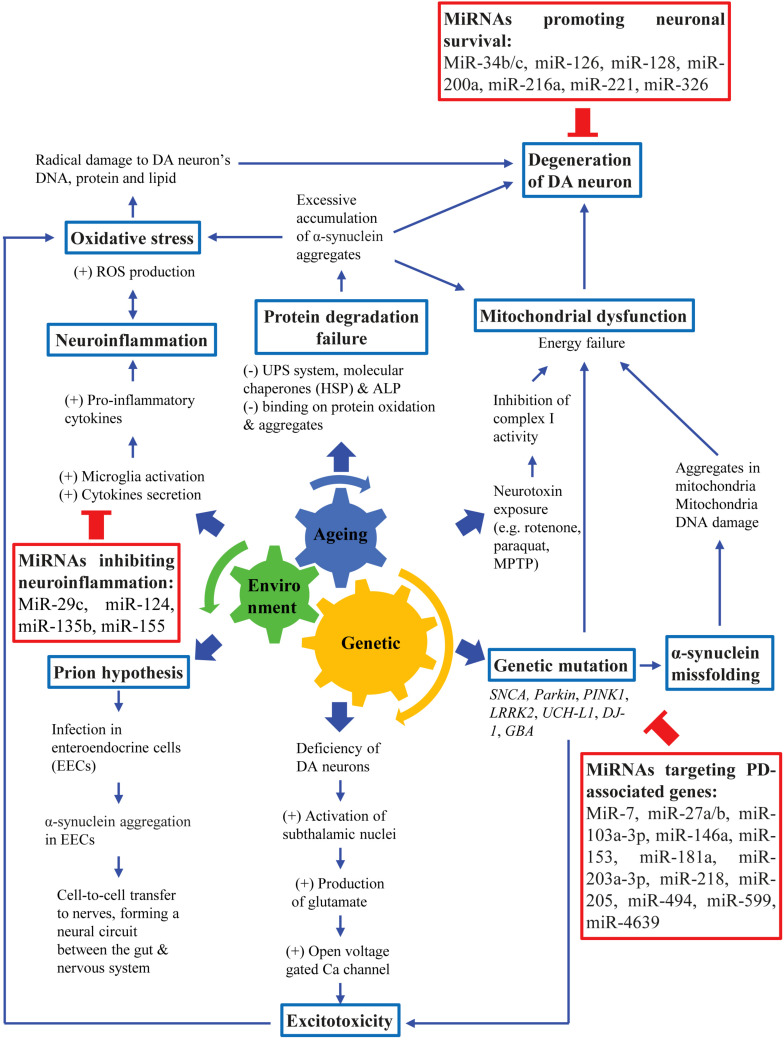
Pathogenesis of PD. PD pathogenesis involves an interplay among aging, genetic susceptibility and environmental factors, leading to loss of DA neurons which eventually lead to cardinal motor symptoms of PD.

The major pathological features of PD are widespread α-synuclein aggregation and progressive degeneration of the nigrostriatal system, leading to the death of DA neurons in the SNpc. This eventually leads to the presentation of both motor and non-motor symptoms of PD (Fujita et al., 2014; Pang et al., 2019; Peball et al., 2020). Additionally, the non-motor symptoms of PD are also mediated by non-dopaminergic systems, and other structures outside the nigrostriatal system (Jellinger, 2017b). PD should be viewed in various aspects in terms of its etiological, molecular biological, anatomical, physiological and pathological aspects of neuropathogenesis. Although the exact etiology of PD is not well understood, different possible causes have been identified. Most PD cases are likely to involve an interplay of aging, genetic susceptibility and exposure to environmental factors (Figure 1; Pang et al., 2019).

Aging is a natural process that involves deregulation of numerous cellular functions and signaling pathways, such as arrested cell cycle, oxidative stress, mitochondrial dysfunction, autophagy and neuro-inflammation that are also implicated in the pathogenesis of neurodegenerative diseases (Dexter and Jenner, 2013; Jellinger, 2017a). Although age is linked to PD, aging itself is insufficient to cause PD. Therefore, it is likely that the association of both genetic and environmental factors are crucial to amplify the SNpc neuronal decline in normal aging to accelerate DA neuronal degeneration as observed in PD patients (Pang et al., 2019). Several PD-causing genes such as α-synuclein (*SNCA*), parkin (*PARK2*), Leucine-rich repeat kinase 2 (*LRRK2*), DJ-1 (*PARK7*) and PTEN-induced kinase 1 (*PINK1*) have been implicated in the pathogenesis of PD and have contributed to the creation of various genetic animal models (Lesage et al., 2020; Tolosa et al., 2020). Additionally, exposure to numerous environmental toxins such as pesticides and heavy metals has been known to increase the risk of PD. Neurotoxins such as 6-hydroxydopamine (6-OHDA), 1-methyl-4-phenyl-1,2,3,6-tetrahydropyridine (MPTP), paraquat and rotenone have been widely used to create PD animal models by selectively destroying DA neurons (Najib et al., 2020).

Although the exact mechanism underlying the death of DA neurons in the SNpc and the presence of Lewy bodies and Lewy neurites remains unclear, mechanisms such as α-synuclein misfolding and their aggregation, mitochondrial dysfunction, oxidative stress, excitotoxicity, impairment of protein clearance pathways and “prion-like protein infection” have been suggested (Golpich et al., 2017; Maiti et al., 2017). The aggregation of α-synuclein has been commonly suggested as the neurodegenerative cause of Parkinsonism (Dickson, 2018). Although most of the α-synuclein immune-reactive pathology in PD is within SNpc and ventral tegmental area, both Lewy bodies and Lewy neurites are also found to be present in nearly all brainstem nuclei and fiber tracts (Seidel et al., 2015). The biological basis for selective vulnerability of DA neurons may be due to the pacemaker-like properties of these neurons that lead to frequent intracellular calcium transients (Guo et al., 2018). Calcium buffering may be relatively deficient in neurons involved in the nigrostriatal pathway compared to neurons in the mesolimbic pathway, which may cause dysregulated cellular homeostasis. DA neuronal death is associated with the loss of functional nuclear envelope integrity and release of pro-aggregant nuclear factors that may then initiate the aggregation of α-synuclein (Jiang et al., 2016). The initiation of aggregation may subsequently lead to spreading to other cells (Braak et al., 2004; Danzer et al., 2012; Dickson, 2018). Degeneration of DA neurons in the SNpc and diminished DA levels in PD patients resulted in less inhibition of striatal neuronal activity and the derangement of striatal neuronal firing. This then led to the loss of inhibitory effects on the globus pallidus and thalamus, which will then cause the excessive activation of the motor cortex. This led to the impairment of motor coordination and patients will exhibit PD-associated motor symptoms (Magrinelli et al., 2016).

## MicroRNAs

MicroRNAs (miRNAs) are a family of small single-stranded non-coding RNA molecules (20 – 25 nucleotides in length) that bind to the 3′ untranslated regions (UTR) of the target mRNAs. The binding induces mRNA degradation and translational repression hence contribute to the post-transcriptional regulation of gene expression. MiRNAs also interact with 5′ UTR, coding sequence, gene promoters and transcription factors (Khee et al., 2014; O’Brien et al., 2018).

The first miRNA, *lin-4* was discovered in 1993 as a developmental gene regulator of *Caenorhabditis elegans*. The *lin-4* contained complementary sequences which enable its binding to the 3′ UTR sequence of *lin-14* (Lee et al., 1993; Wightman et al., 1993). The binding of *lin-4* to the *lin-14* forms multiple RNA duplexes that lead to the down-regulation of *lin-14* translation (Wightman et al., 1993). To date, around 48,860 mature miRNAs have been identified from 271 different species, including over 2,600 miRNAs from humans, and these data are registered in miRBase (the miRNA database^1^) (Kozomara et al., 2019).

The identification of target genes is important to understand the function of miRNAs. Currently, numerous bioinformatics and biological tools are used to identify miRNA target genes, which may then be validated with further experiments (Wang et al., 2017).

## MicroRNA Dysregulation in Parkinson’s Disease

Dysregulation of miRNAs will result in development and progression of numerous disease, such as in cancers (Ibrahim et al., 2015). Studies have identified dysregulation of miRNAs expression in neurodegenerative diseases such as Alzheimer’s disease, amyotrophic lateral sclerosis and Huntington’s disease (Lee et al., 2011; Figueroa-Romero et al., 2016; Putteeraj et al., 2017). Similarly, in PD patients, the striatal brain tissue and DA neurons from the SNpc demonstrated dysregulated miRNAs expression profiles (Briggs et al., 2015; Nair and Ge, 2016). A total of 125 miRNAs was significantly altered in the post-mortem analysis of the prefrontal cortex from PD patients compared to the neuropathologically normal controls (Hoss et al., 2016). Therefore, it is believed that miRNA dysregulation may contribute to the pathogenesis of PD by regulating various genes and proteins related to PD. This review will discuss the recent findings of miRNAs dysregulation related to PD.

### MiRNAs Related to the Regulation of PD-Associated Genes (Table 1)

#### SNCA: miR-7, miR-153 and miR-203a-3p

MiR-7, miR-153 and miR-203a-3p possess binding sites to the 3′ UTR of the *SNCA* gene. The expression of miR-7 is significantly reduced in the SNpc of PD patients (McMillan et al., 2017). It has been shown that over-expression of both miR-7 and miR-153 in the human embryonic kidney cell line, HEK293 cells and cortical neurons lead to significant reduction of *SNCA* mRNA and protein expression levels meanwhile miR-7 knockdown induces over-expression of α-synuclein protein level (Doxakis, 2010; McMillan et al., 2017). Both miRNAs regulate SNCA protein levels via a different pathway in which miR-7 inhibits its translation while mir-153 degrades the mRNA (Doxakis, 2010). Je and Kim (2017) demonstrated that exposure of 1-methyl-4-phenylpyridinium (MPP^+^, the active metabolite of MPTP) to the HEK293 cells relieved the miRNA-mediated translational suppression of *SNCA*, via MPP^+^-mediated mitochondrial reactive oxygen species (ROS) production. Interestingly, the over-expression of miR-7 and miR-153 in cortical neurons reduced MPP^+^-induced neurotoxicity by increasing neuronal viability, up-regulation of pro-survival BCL-2 protein and in-activation of pro-apoptotic caspase-3 (Fragkouli and Doxakis, 2014).

**TABLE 1:** Expression of miRNAs targeting PD-related genes.

**Target**	**miRNA**	**Expression compared to control**
SNCA	miR-7	–
	miR-153	↑
PRKN	miR-181a	↓
LRRK2	miR-205	↑
DJ-1	miR-494	–

Another miRNA targeting *SNCA*, miR-203a-3p expression was down-regulated in MMP^+^-treated human dopaminergic neuroblastoma, SH-SY5Y cells (Jiang et al., 2020). MMP^+^ treatment led to reduce cell proliferation and induced apoptosis in the SH-SY5Y cells that then led to enhance expression of SNCA, p53 and cleaved Caspase-3 proteins of which were inhibited by the up-regulation of miR-203a-3p (Jiang et al., 2020). Other miRNAs that were identified as regulators of SNCA expression include the miR-30b, miR-34b/c, miR-214, and miR-433 (Wang et al., 2015; Consales et al., 2018; Tarale et al., 2018; Shen et al., 2020).

#### *PRKN*: miR-103a-3p, miR-146a, miR-181a and miR-218

Mutations in the *PRKN*, encoding Parkin (an important E3 ubiquitin-protein ligase that mediates the elimination of damaged mitochondria via mitophagy), cause an autosomal recessive form of early onset PD (Kim et al., 2012; Zanellati et al., 2015). Mitochondrial dysfunction and the down-regulation of Parkin in the MPTP-treated mice and MPP^+^-treated SH-SY5Y cells (Zhou et al., 2020). Furthermore, Parkin protein expression was significantly down-regulated following miR-103a-3p over-expression while Parkin protein expression was up-regulated following miR-103a-3p knockout. This suggest that miR-103a-3p able to regulate Parkin expression, by binding to the 3′-UTR of Parkin mRNA (Zhou et al., 2020).

In a rotenone-induced PD rat model, miR-146a, a known inflammation regulatory miRNAs was identified as the most up-regulated miRNAs (Jauhari et al., 2020). The exposure of rotenone led to the activation of NF-κβ and induces the transcription of miR-146a that is responsible for the down-regulation of Parkin protein level (Jauhari et al., 2020). In contrast, down-regulation of miR-146a expression led to the inhibition of NF-κβ phosphorylation and increased Parkin level in the rotenone-treated SH-SY5Y cells (Jauhari et al., 2020).

*PRKN* was also identified as a target of other miRNAs such as miR-181a and miR-218. Over-expression of miR-181a significantly reduced Parkin mRNA and protein level, while inhibition of miR-181a increased Parkin expression (Cheng et al., 2016). In addition to *PRKN*, miR-181a also targets genes that control neurite growth namely *SMAD1* and *SMAD5* transcription factors (Hegarty et al., 2018). Inhibition of miR-181a expression in SH-SY5Y cells led to elevation of Smad1/5 proteins, as well as the neurite length and neurite branching (Hegarty et al., 2018). Similarly, down-regulation of miR-181a-5p confers neuroprotection to MPP^+^-treated cells by enhancing cell viability and suppression of cell apoptosis, LDH activity and inflammation (Guo and Hua, 2020). The data on miR-181a dysregulation reported in PD patients and axonal degeneration of DA neurons in early stages of PD suggest that inhibition of miR-181a may be a promising therapeutic approach for PD. MiR-218 also targets *PRKN* and negatively regulates PINK1/PRKN-mediated mitophagy. Over-expression of miR-218 down-regulated PRKN expression, leading to dysregulated mitophagy (Di Rita et al., 2020). However, miR-218 expression was found to be reduced in the patients and animal models (Xing et al., 2020; Ma et al., 2021).

#### *PINK1*: miR-27a/b

Homozygous and compound heterozygous mutations in the *PINK1* gene are associated with the early onset of PD (Krohn et al., 2020). The *PINK1* gene encodes a PTEN-induced serine-threonine kinase 1 located in the mitochondria in which the mutations can lead to respiratory chain dysfunction and impairment in ATP production (Rango et al., 2020). MiR-27a and miR-27b suppressed the expression of PINK1 by direct binding to the 3′UTR of its mRNA (Kim J. et al., 2016). Thus, miR-27a/b inhibited accumulation of PINK1, which in turn prevented the translocation of Parkin to the mitochondria following mitochondrial damage hence leads to the inhibition of lysosomal degradation of damaged mitochondria (Kim J. et al., 2016). In addition to miR-27a/b, miR-140 has also been reported to target PINK1. Amyloid β-derived diffusible ligands-induced neurons transfected with miR-140 mimic exhibited up-regulated miR-140 and down-regulated PINK1 expressions. Reciprocal effects were seen following miR-140 inhibitor transfection (Liang et al., 2021).

#### *LRRK2*: miR-205 and miR-599

The *LRRK2* gene mutations are associated with familial and sporadic forms of PD (Gopalai et al., 2014). The LRRK2 protein level increased with no significant changes in its mRNA levels in the frontal cortex of sporadic PD patients hence suggesting the involvement of miRNAs in suppressing post-transcriptional regulation of LRRK2 (Cho et al., 2013). There were reduced miR-205 expression in PD patients, and the reduction showed inverse correlation between the expression of LRRK2 protein and miR-205 (Cho et al., 2013). Similarly, over-expression of miR-205 in HEK293T cells suppressed LRRK2 protein expression *in vitro*, while inhibition of miR-205 enhanced LRRK2 protein expression (Cho et al., 2013).

MiR-599 expression was down-regulated both *in vivo* [lipopolysaccharide (LPS) administered mice] and *in vitro* (MPP^+^-treated SH-SY5Y cells) PD models, which was associated with increased LRRK2 expression (Wu et al., 2019). Over-expression of miR-599 was shown to down-regulate LRRK2 expression while knockdown of miR-599 up-regulated LRRK2 expression in the MPP^+^-treated SH-SY5Y cells (Wu et al., 2019).

#### *PARK7*: miR-494 and miR-4639

DJ-1 (*PARK7*) is identified as a recessive familial PD gene in which loss of this gene resulted in the increased the susceptibility of cells to the damaging effect of oxidative stress, leading to the early onset of PD (Irrcher et al., 2010). MiR-494 binds to the 3′ UTR of *DJ-1* and leads to the inhibition of *DJ-1* transcription (Xiong et al., 2014). Over-expression of miR-494 in 3T3 cells resulted in decreased DJ-1 protein level and increased ROS production without changing the mRNA expression (Xiong et al., 2014). Similarly, over-expression of miR-494 in mice leads to a decrease in *DJ-1* expression in the SNpc following MPTP administration, alongside the exacerbated degeneration of DA neurons in the SNpc and significant motor impairment (Xiong et al., 2014; Geng et al., 2018).

The miR-4639-5p level was up-regulated in the plasma of PD patients whereby miR-4639-5p negatively regulates *DJ-1* in the post-transcriptional level (Chen et al., 2017). The up-regulation of miR-4639-5p led to the down-regulation of DJ-1 protein level hence resulted in severe oxidative stress and neuronal death in MPP^+^- and rotenone-treated SH-SY5Y cells (Chen et al., 2017). MiR-145-3p and miR-874 are among other miRNAs that were predicted to regulate DJ-1 expression, and both were shown to be highly expressed in the saliva of PD patients (Chen et al., 2020).

### MiRNAs Related to Dopaminergic Neurons Survival

#### MiR-34b/c

The expression of both miR-34b and miR-34c were shown to be down-regulated in various brain region of PD patients including the putamen, amygdala, substantia nigra, frontal cortex, and cerebellum (Minones-Moyano et al., 2011; Villar-Menendez et al., 2014). Inhibition of miR-34b/c expression in SH-SY5Y cells lead to reduced cell viability, mitochondrial dysfunction and increased ROS generation in the cells (Minones-Moyano et al., 2011). In addition, miR-34b/c inhibition could also lead to a significant reduction in DJ-1 and Parkin protein levels in which these proteins are associated with familial forms of PD (Minones-Moyano et al., 2011). In another study, miR-34b/c inhibition increased the α-synuclein levels and stimulate the aggregate formation, possibly contributing to PD pathogenesis (Kabaria et al., 2015).

#### MiR-126

MiR-126 was up-regulated in the SNpc DA neurons from PD patients (Kim W. et al., 2014). Over-expression of miR-126 compromised the survival of primary DA neurons and SH-SY5Y cells hence increasing their vulnerability to 6-OHDA toxicity (Kim W. et al., 2014). In addition to DA neurotoxins, miR-126 over-expression also increases neuronal vulnerability to staurosporine (a non-selective protein kinase inhibitor) and Alzheimer’s disease-associated amyloid β 1-42 peptides (Kim W. et al., 2016). The miR-126 over-expressing cells showed reduced expression of p85β, IRS-1 and SPRED1, reduced phosphorylated AKT and extracellular signal-regulated kinase (ERK) protein levels. These results suggest that miR-126 down-regulates insulin/IGF-1/phosphatidylinositol-3-kinase (PI3K)/AKT and ERK signaling cascades in promoting neuronal death in PD (Kim W. et al., 2014).

#### MiR-128

The expression of miR-128 decreased in the MPTP-induced PD mice (Zhou et al., 2018; Zhang G. et al., 2020). MiR-128 protects DA neurons from apoptosis and up-regulates the expression of Excitatory Amino Acid Transporter 4 (EAAT4) by binding to the axis inhibition protein 1 (AXIN1) (Zhou et al., 2018). Further study reported that the increased HIF-1α/miR-128-3p inhibited apoptosis of hippocampal neurons through Wnt/β-catenin signaling pathway activation via the suppression of AXIN1 (Zhang G. et al., 2020).

#### MiR-200a

The expression of miR-200a was significantly up-regulated in the pheochromocytoma cell line PC12 cells and SH-SY5Y cells following exposure to MPP^+^ that leads to the induction of oxidative stress and cell apoptosis (Salimian et al., 2018; Talepoor Ardakani et al., 2019). Additionally, the transcript level of SIRT1 (a miR-200a target) showed significant down-regulation in the MPP^+^-treated cells, confirming that SIRT1 may induce DA neuronal apoptosis via P53 and FOXO signaling pathways (Salimian et al., 2018). In contrast, inhibition of miR-200a reduced the apoptosis and increased DA neuronal population in 6-OHDA-treated PD rats, via inhibition of the cAMP/PKA signaling pathway (Wu et al., 2018).

#### MiR-216a

MiR-216a over-expression has shown to provide protection against MPP^+^-induced neurotoxicity in SH-SY5Y cells by significantly increase neuronal viability following MPP^+^ treatment (Yang et al., 2020). This was also associated with reduced neuronal apoptosis, ROS level and lipid peroxidation (Yang et al., 2020). MiR-216a promotes neuronal survival by down-regulating Bax that is involved as apoptotic regulators (Yang et al., 2020). The up-regulation of miR-216a also exerts its neuroprotective effects against ischemic brain injury by targeting JAK2/STAT3 pathways (Tian et al., 2018).

#### MiR-221

MiR-221 expression was significantly down-regulated in 6-OHDA-treated PC12 cells (Li et al., 2018). Over-expression of miR-221 cells treated with 6-OHDA and MPP^+^ significantly promotes cell viability and proliferation while inhibited cell apoptosis (Li et al., 2018; Oh et al., 2018). The brains of DJ-1^–/–^ mouse and DJ-1 knock down SH-SY5Y cells demonstrated down-regulation of miR-221 (Oh et al., 2018). Additionally, miR-221 down-regulated the expression of pro-apoptotic proteins (bcl-2-like protein 11, bcl2 modifying factor, and bcl2 interacting protein 3-like) at basal conditions thus prevented the oxidative stress-induced increase of bcl-2-like protein 11 (Oh et al., 2018). Based on these findings, the modulation of miR-221 by DJ-1 could contribute to the pathogenesis of autosomal recessive inherited PD or sporadic PD, resulted from the loss-of-function mutations in DJ-1.

#### MiR-326

The expression of miR-326 was shown to be down-regulated in MPTP-induced PD mice (Zhao et al., 2019). MiR-326 over-expression ameliorates the motor dysfunction and the structural abnormality of the SNpc (increase content of DA, SOD, GSH-Px, and TH positive expression) in PD mice (Zhang et al., 2019). In addition, miR-326 overexpression also inhibits numerous immunomodulatory factors such as TNF-α, IL-1, IL-6, and INF-γ. Mice treated with miR-326 mimic inhibits expression of iNOS and neuronal apoptosis and promotes autophagy of DA neurons (Zhang et al., 2019; Zhao et al., 2019). Recent evidence also showed that miR-326 may play an important role as the key suppressor in the development of PD due to the suppression of pyroptotic cell death with the activation of NLRP3 inflammasome. It was shown that silencing the lncRNA homeobox transcript antisense RNA resulted in significant inhibition of neuronal damage through the suppression of NLRP3-mediated pyroptosis via the regulation of miR-326/ELAVL1 axis in PD model (Zhang Q. et al., 2020).

### MiRNAs Related to Neuroinflammation

#### MiR-29c

The serum miR-29 level was significantly down-regulated in PD patients, and were correlated with the disease severity (Bai et al., 2017). Over-expression of miR-29c attenuated DA neuronal death and α-synuclein aggregation in the SNpc of MPTP-treated mice. Furthermore, miR-29c exhibited anti-inflammatory properties by ameliorated the elevated pro-inflammatory cytokines (TNF-α, IL-1β, and IL-6) and apoptosis in the MPTP-treated mice (Wang et al., 2020b). Additionally, over-expression of miR-29c suppressed the LPS-induced microglial activation, via modulation of NOD-like receptor protein 3 (NLRP3) inflammasome by targeting nuclear factor of activated T-cells (NFAT) 5 (Wang et al., 2020a).

#### MiR-124

The expression of miR-124 decreased in the midbrain of the MPTP-induced PD mouse model (associated with reduced DA neurons, and decreased tyrosine hydroxylase and DA transporter expressions), MPP^+^-treated SH-SY5Y and MN9D cells (Kanagaraj et al., 2014; Liu et al., 2017). The reduced miR-124 expression increased the expression of calpain 1, leading to the activation of the p25/cdk5 pathway, which is known to be involved in the DA neuronal loss in PD (Kanagaraj et al., 2014). Additionally, miR-124 suppression increased cell autophagy-related protein expression (LC3 II and Beclin 1), increased phosphorylated-AMPK level while decreased p-mTOR expression suggest that miR-124 regulates DA neuronal apoptosis and autophagy by regulating the AMPK/mTOR pathway (Gong et al., 2016). In contrast, up-regulation of miR-124 through administration of miR-124 in the MPTP-treated mice attenuated the loss of DA neurons and striatal DA level caused by MPTP toxicity (Wang H. et al., 2016). Additionally, over-expression of miR-124 protects SH-SY5Y cells from cell death caused by microglial activation following LPS exposure suggesting the miR-124 role in the inhibition of neuroinflammation (Yao et al., 2018). In addition, over-expression of miR-124 effectively attenuated the expression of pro-inflammatory cytokines (iNOS, IL-6, and TNF-α) and promote the secretion of neuroprotective factors (TGF-β1 and IL-10) in LPS-induced immortalized murine microglial cell line BV2 cells (Yao et al., 2018). Further study suggested that miR-124 inhibits neuroinflammation by targeting sequestosome 1 (p62), phospho-p38 mitogen-activated protein kinases and autophagy (Yao et al., 2019).

#### MiR-135b

Exposure of MPP^+^ to SH-SY5Y cells leads to a significant down-regulation of miR-135b (Zhang et al., 2017). Over-expression of miR-135b was able to attenuate neurotoxicity effects of MPP^+^: increased in cell viability, suppressed apoptosis, reduced level of cleaved caspase 3 (an apoptotic marker), and inhibited MPP^+^-induced TNF-α and IL-1β secretion (Zhang et al., 2017). Over-expression of miR-135b suppressed MPP^+^-induced increased quantity of pyroptotic cells and protein levels of various pyroptotic related genes (TXNIP, NLRP3, Caspase-1, ASC, GSDMD^Nterm^ and IL-1β) in the SH-SY5Y and PC-12 cells (Zeng et al., 2019).

#### MiR-155

MiR-155 demonstrated enhanced expression in the SNpc of PD mice model produced by adeno-associated-virus-mediated expression of α-synuclein (Thome et al., 2016). The deletion of miR-155 in mice prevents α-synuclein-associated neurodegeneration of DA neurons, which corresponds to the reduced microgliosis (Thome et al., 2016). Prolong exposure of TNF-α (PD patients demonstrated increased TNF-α level in the serum and CSF) to SH-SY5Y cells induced apoptosis, dysregulation of complex I and mitochondrial oxidative stress (Prajapati et al., 2015). TNF-α exposure also resulted in the up-regulation of numerous miRNAs, including miR-155, which may target nuclear encoded subunits of mitochondrial complex-I (NADH:Ubiquinone Oxidoreductase Core Subunit V1) (Prajapati et al., 2015). In addition, DJ-1 deficiency increased the expression of miR-155 in microglia and astrocytes, which down-regulates the expression of suppressor of cytokine signaling 1 (SOCS1) (Kim J. H. et al., 2014). Taken together, miR-155 modulates microglia-mediated inflammation by down-regulating SOCS1 expression and increasing the production of cytokine and nitric oxide. This leads to inefficient termination of STAT1-induced inflammatory response, which may cause neuronal death (Cardoso et al., 2012; Kim J. H. et al., 2014).

## MiRNAs Dysregulation in Induced Pluripotent Stem Cell-Based PD Modeling

Disease modeling using induced pluripotent stem cell (iPSC) has shown to be useful by providing cellular insight into disease pathology in various neurodegenerative diseases, including PD (Doss and Sachinidis, 2019). For example, iPSC-derived DA neurons from sporadic PD and monogenic LRRK2-associated PD patients exhibited global DNA hyper-methylation changes (Fernandez-Santiago et al., 2019). An explorative genome-wide study of miRNAs expression levels in iPSC-derived DA neurons from PD patients identified an up-regulation of 5 miRNAs (miR-9-5p, miR-135a-5p, miR-135b-5p, miR-449a, and miR-449b-5p) and down-regulation of 5 miRNAs (miR-141-3p, miR-199a-5p, miR-299-5p, miR-518e-3p, and miR-519a-3p) (Tolosa et al., 2018). The genes targeted by the identified miRNAs were involved in regulating cytoskeleton, axonal transport, cell adhesion and cell survival in PD.

## Circulating MiRNAs as Diagnostic Biomarkers for PD

Circulating miRNAs are secreted from cells and have been found in various biological fluids, such as plasma, serum, saliva, milk, urine, seminal plasma, amniotic fluid, cerebrospinal fluid (CSF), peritoneal fluid, and pleural fluid (Turchinovich et al., 2012; Faruq and Vecchione, 2015). Extracellular miRNAs can either be contained within membranous vesicles, or are associated with Argonaute proteins (Turchinovich et al., 2012). The circulating miRNAs are stable in different conditions as they are resistant to RNases activity, high or low pH, multiple freeze-thaw cycles and long-term storage at room temperature thus making them suitable to be used as disease biomarkers (Turchinovich et al., 2012; Makarova et al., 2015). Various studies have described the use of circulating miRNAs as non-invasive biomarkers for the diagnosis of different diseases, including cancers, liver diseases, respiratory diseases, kidney diseases, and myocardial infarction (Arrese et al., 2015; Tiberio et al., 2015; Coskunpinar et al., 2016; Huttenhofer and Mayer, 2017; Teoh and Das, 2017; Pattarayan et al., 2018).

Circulating miRNAs profile has been analyzed from CSF, serum, plasma, peripheral blood mononuclear cells (PBMCs), and saliva collected from PD patients (summarized in Table 2; Gui et al., 2015; Ding et al., 2016; Ma et al., 2016; Cao et al., 2017; Li et al., 2017; Marques et al., 2017; Schwienbacher et al., 2017; Caggiu et al., 2018; Chen et al., 2018; Yang et al., 2019b; Cressatti et al., 2020; Ozdilek and Demircan, 2020; Xie et al., 2020). These studies have identified numerous miRNAs as potential PD biomarkers. In an example, the expression of miR-144-5p, miR-200a-3p, and miR-542-3p that were dysregulated in A53T mutant α-synuclein transgenic mice, were also shown to be up-regulated in the CSF of PD patients (Mo et al., 2017). Additionally, the reduced CSF miR-626 and elevated plasma miR-105-5p levels may be able to be used as a biomarker to differentiate PD from other neurodegenerative diseases, for example, Alzheimer’s disease (Yang et al., 2019a; Qin et al., 2021). Furthermore, apart from identifying PD, circulating miRNAs such as miR-29a, miR-29c, miR-30c-5p, miR-132-3p, miR-146a-5p, and miR-373 were found to be correlated with the severity of PD (Bai et al., 2017; Shu et al., 2020; Zhang L. et al., 2020).

**TABLE 2:** Circulating miRNA profile from PD patients.

**Samples**	**Analysis method**	**miRNA expression in PD**		**References**
		**Up-regulation**	**Down-regulation**	
CSF	qRT-PCR	–	miR-626	Qin et al. (2021)
	NGS	Let-7f-5p	miR-27a-3p, miR-423-5p	Dos Santos et al. (2018)
	qRT-PCR	miR-205	miR-24	Marques et al. (2017)
	qRT-PCR	miR-144-5p, miR-200a-3p and miR-542-3p	–	Mo et al. (2017)
	TaqMan low-density array human miRNA panels	let-7c-3p, let-7g-3p, miR-10a-5p, miR-16-2, miR-26a, miR-30b, miR-103a, miR-127-3p, miR-132a-5p, miR-136-3p, miR-153, miR-370, miR-409-3p, miR-433, miR-485-5p, miR-873-3p	miR-1, miR-19b-3p, miR-22, miR-28, miR-29, miR-29c, miR-119a, miR-126, miR-151, miR-301a, miR-374	Gui et al. (2015)
Serum	qRT-PCR	miR-29c	–	Ozdilek and Demircan (2020)
	qRT-PCR	–	miR-132-3p, miR-146-5p	Shu et al. (2020)
	qRT-PCR	miR-30c-5p, miR-373	–	Zhang L. et al. (2020)
	qRT-PCR	–	miR-29	Bai et al. (2017)
	qRT-PCR	miR-24, miR-195	miR-19b	Cao et al. (2017)
	qRT-PCR	–	miR-29c, miR-146a, miR-214, miR-221	Ding et al. (2016)
	Solexa sequencing followed by qRT-PCR	miR-195	miR-15b, miR-181a, miR-185, miR-221	Ma et al. (2016)
	Solexa sequencing followed by qRT-PCR	-	miR-141, miR-146b-5p, miR-193a-3p, miR-214	Dong et al. (2016)
Plasma	qRT-PCR	miR-105-5p	–	Yang et al. (2019a)
	qRT-PCR	miR-132	–	Yang et al. (2019b)
	qRT-PCR	miR-27a	Let-7a, let-7f, miR-142-3p, miR-222	Chen et al. (2018)
	qRT-PCR	miR-137	miR-124	Li et al. (2017)
Plasma and WBC	qRT-PCR	miR-30a-5p	–	Schwienbacher et al. (2017)
Plasma extracellular vesicles	qRT-PCR	miR-30c-2-3p	miR-15b-5p, miR-106b-3p, miR-138-5p, miR-338-3p, miR-431-5p	Xie et al. (2020)
PBMC	qRT-PCR	miR-155-5p	miR-146a-5p	Caggiu et al. (2018)
Saliva	qRT-PCR	–	miR-153, miR-223	Cressatti et al. (2020)
	qRT-PCR	miR-145-3p, miR-874	–	Chen et al. (2020)

The accurate diagnosis of PD is difficult, especially in its early stages. PD is normally diagnosed with the presence of motor symptoms in which loss of more than 50% DA neurons were reported in PD patients (Kordower et al., 2013). Early detection and monitoring of PD would ensure that the available treatment could potentially slow down the progression of the disease. Dos Santos et al. (2018) demonstrated that CSF obtained from patients in the early stage of PD express increased Let-7f-5p level and reduced miR-27a-3p and miR-423-5p levels. The healthy controls, on the other hand, express high levels of miR-125a-5p and low levels of miR-151a-3p in the CSF. Alternatively, serum expression of miR-141, miR-214, miR-146b-5p, and miR-193a-3p should be reduced in the early stage of PD patients compared to the controls (Dong et al., 2016). Another recent study showed that a compound down-regulation of *SRRM2* and miR-27a-3p with an up-regulation of miR-27b-3p in PBMCs of PD patients is associated with the early stage onset of the disease (Fazeli et al., 2020). Additionally, circulating miRNAs also differ between early-onset and late-onset PDs. Sulaiman et al. (2020) reported a total of 5 miRNAs were found in early-onset PD patients in which 1 miRNA was up-regulated (miR-29b-3p) and 4 miRNAs were down-regulated (miR-297, 4462, 1909-5p, and 346) and the predicted targets of these miRNAs are involved in DA synapse regulation.

Despite the promising results from previous studies showing the potential of miRNAs as PD biomarkers, there are limited overlapping miRNAs identified across different studies, even from the same biological fluid. This was observed in 7 studies analyzing serum obtained from PD patients as shown in Table 1, miR-195 was found to be up-regulated in only 2 studies, while miR-146-5p, miR-214, and miR-221 were down-regulated as demonstrated in another 2 studies, suggesting the low reproducibility of these miRNAs as a disease biomarker. To increase the specificity and reproducibility of miRNAs as potential PD biomarkers, methods of miRNAs detection in samples and adequately quality-controlled data processing need to be standardized in future studies (Wang J. et al., 2016). Additionally, to increase the accuracy of miRNAs as a biomarker, Patil et al. (2019) demonstrated the use of a combination of serum miRNAs such as miR-335-5p/miR-3613-3p, miR-335-5p/miR-6865-3p, and miR-335-5p/miR-3613-3p/miR-6865-3p that showed a high degree of discriminatory accuracy in a large cohort.

## MiRNAs as Novel Therapeutic Tools for PD

Since miRNAs can regulate multiple genes, they may serve as potential therapeutic tools for PD. MiRNAs can be used to silence up-regulated genes in PD, while anti-miRNAs can be used to restore a down-regulated target gene. To overcome the issue of instability due to enzyme degradation, consideration of miRNA-based therapeutics would require a safe and stable delivery system to protect the miRNAs from degradation and facilitate their ability to cross the blood-brain-barrier (Wen, 2016).

Previous studies have demonstrated decreased miR-124 expression in various *in vivo* and *in vitro* PD models, whereas administration of miR-124 was able to reverse the loss of DA neurons and inhibit neuroinflammation in the MPP^+^-treated SH-SY5Y cells, MPP^+^-treated MN9D cells and MPTP-treated mice (Wang H. et al., 2016; Yao et al., 2018, 2019). Polymeric nanoparticles which are stable in systemic circulation can be used to deliver miR-124 to a specific region of the brain. Rabies virus glycoprotein (RVG29)-linked miR-124-loaded polymeric nanoparticles were able to cross through the *in vitro* blood-brain-barrier model (Gan et al., 2019). Similarly to the *in vitro* study, administration of the miR-124-loaded polymeric nanoparticles into the lateral ventricle of the MPTP-administered mice was able to inhibit Mitogen activated protein kinase kinase kinase-3 (MEKK3 which plays a critical role in mediating NF-κB activation) signaling and the activation of microglia (Gan et al., 2019).

As PD is also characterized by loss of DA neurons in the SNpc, enhancing adult neuroregeneration (generation of functional neurons from adult neural precursors) could be one therapeutic approach to treat PD. In mammals, adult neurogenesis occurs throughout life in restricted brain regions, which include the dentate gyrus of the hippocampus and from the subventricular zone of the lateral ventricle (Ming and Song, 2011). miR-124 loaded nanoparticles administered to the subventricular zone cells primary culture did not affect overall cell proliferation. However, this treatment increased neuroblast proliferation while decreasing the proliferation of astrocytic-like cells, by repressing the expression of non-neuronal genes (*Sox9* and *Jagged1*) (Saraiva et al., 2016). Intraventricular administration of miR-124 loaded nanoparticles *in vivo* increased the number of subventricular zone-derived neuroblasts in the striatum of 6-OHDA-treated mice. This data support the potential of miRNAs as therapeutic strategies to induce neuroregeneration and to promote brain repair (Saraiva et al., 2016).

## Conclusion

This review reported that the dysregulation of several miRNAs has a significant role in the pathogenesis of PD. These miRNAs can be classified into those which are involved in the regulation of PD-associated genes, DA neuronal health and α-synuclein-induced neurodegeneration. Understanding the roles of these miRNAs not only shed some light on the pathogenesis of PD, but could also serve as potential therapeutic targets since regulating specific microRNAs was found to have beneficial effects in the *in vitro* and *in vivo* models of PD. Further studies are therefore essential to explore further functions of these miRNAs as well as others that are yet to be discovered in the pathogenesis and therapeutic options of PD.

## Author Contributions

SLT planned the conceptual ideas and proof outline. YHN, NHMN, MAK, and SLT wrote the manuscript. WLL, MFY, and SLT critically revised the manuscript. All authors contributed to the article and approved the submitted version.

## Conflict of Interest

The authors declare that the research was conducted in the absence of any commercial or financial relationships that could be construed as a potential conflict of interest.

## Footnotes

1 http://www.mirbase.org/
